# A Letter on the Suppression of Quack Medicines

**Published:** 1799-09

**Authors:** 


### *To the Editors of the Medical and Physical Journal.*

Gentlemen,

A very sensible letter appeared in the fourth Number of the Medical and Physical Journal, page 337, on the subject of Quackery. It is much to be wished, that some such regulations as are therein suggested, might be adopted to suppress the baneful compositions of the ignorant and daring self-created doctors, or at least to prevent their so very general circulation and use.

There is, I fear, little reason to expect that the College of Physicians, whatever powers they are possessed of, will interest themselves in the affair; but without their assistance, much may be effected by the profession at large, if they will only consent to oppose the quacks with their own weapons.

It is evident, that the only way a quack-medicine gets very celebrated, is, by its being constantly puffed off in advertisements. Whenever a cure has been performed, or supposed to have been performed by any of these compositions, thousands of advertisements are distributed in all possible ways, to make it universally known and attended to; but not a word is whispered, not a syllable dropt, of the thousands who, having credulously swallowed these incongruous remedies, have found the relief *they* could not give, in the grave, or have continued to exist miserably, in a state worse than death! Why are not these cases made public? What should hinder professional men, who are daily witnessing the mischiefs occasioned by these noxious drugs, from publishing them to the world? The expence, I conceive, might might eaſily be defrayed by ſubſcription (which I have little doubt would be ſoon filled up), and it is to be hoped, that in theſe caſes, truth would not be eſteemed a libel *.

In addition to this, it would be proper to endeavour by occaſional addreſſes in the public papers, to convince the reſpectable perſons, who are ſometimes prevailed upon to atteſt cures, ſaid to be performed by theſe medicines, of the injuries they may occaſion by ſuch injudicious conduct, though their motives are undeniably praiſe-worthy; for though they may believe that the remedies recommended, are very proper in caſes ſimilar to thoſe, in which they have experienced good effects, yet as ſick perſons and their friends are very frequently, and very woefully miſtaken, in their opinions of diſeaſes, the uſe of medicines thus recommended, may be attended with fatal effects.

The names of dignitaries in the church and law have, for ſome months, been pompouſly exhibited upon all the poſts and dirty walls of the metropolis, as recommenders of ſome newly-broached worm-medicines; but ſurely, theſe great and eſtimable characters would never have ſo publicly ſanctioned theſe remedies, however uſeful they may prove as anthelmintics, were they aware, that children are often ſuſpected to have worms where none exiſt, and that under ſuch circumſtances, theſe medicines may do infinite miſchief; of which the following is an inſtance:

A few months ago, a child about three years of age, was ſuſpected by his parents to have worms; without aſcertaining this, they determined to give the medicines thus trumpeted forth as ineſtimable by the Proprietor, and ſanctioned by ſuch high names. The firſt doſe, given according to the printed directions, made the child very ill; not diſcouraged, however, they gave a ſecond and third, and then the patient was ſo alarmingly worſe, as made his friends deſirous of regular advice. The phyſician who had on former occaſions attended the family, was called in, and found the child languid and weak, the pulſe quick and low, the lips pale, appetite gone, breath offenſive, breathing ſhort, frequent, and profuſe, hæmorrhage from the noſe, and ſmall purple ſpots appearing on all parts of the body. This exceſſive ſtate of debility appeared to have been produced by the injudicious exhibition of theſe powerfully draſtic purgatives, to a child who was ill in conſequence of long-continued cold, damp weather, and poor diet. Had this child, inſtead of being treated for worms (in which caſe theſe medicines

* Some correspondent may, perhaps, give proper information on this subject, as it is understood, that Dr. LETTSOM, who so spiritedly pointed out the injurious effects of a late Quack's nostrums, was prosecuted for a libel, and obliged to pay damages.

cines would have been much too rough for him), taken ſome light cordials, joined with gentle purgative phyſic, and been ſupplied with proper diet, he might, probably, have been now alive; as it was, though partially recovered by proper means from the ſtate above deſcribed, he gradually ſunk into an atrophy, and died completely exhauſted.

Societies are forming almoſt every day for various beneficial purpoſes; let one, then, equally beneficial, be formed for the purpoſe of convincing the public of the dangerous conſequences of quackery. To this end, let well-atteſted caſes be publiſhed of the injuries produced by the uſe of theſe unknown remedies; no unfair means ſhould be uſed, but every well-authenticated failure ſhould be fairly and unequivocally ſubmitted to the public.

If the public are in this manner put upon their guard, it is to be imagined that laws and regulations to reſtrain quacks will be leſs neceſſary; ſomething effectual, however, in that way, ſhould be attempted, and a ſociety inſtituted for the aforementioned purpoſe, would probably ſoon be able to furniſh a plan effectual for this.

*July* 12, 1799. S. M.

---

